# Recognizing ALBI Grade in Child-Pugh A Patients at a Glance: Mathematical Simulation and Large-Scale Clinical Validation

**DOI:** 10.3390/diagnostics16030370

**Published:** 2026-01-23

**Authors:** Po-Heng Chuang, Yuan-Jie Ding, Chih-Yun Lin, Sheng-Nan Lu

**Affiliations:** 1 Division of Gastroenterology and Hepatology, Department of Internal Medicine, Jen-Ai Hospital, Dali Branch, Taichung 41265, Taiwan; 2 College of Health Sciences, Central Taiwan University of Science and Technology, Taichung 406053, Taiwan; 3 Division of Gastroenterology and Hepatology, Department of Internal Medicine, Chang Gung Memorial Hospital, Chiayi 613, Taiwan; 4 Biostatistics Center, Kaohsiung Chang Gung Memorial Hospital, Kaohsiung 833, Taiwan; 5 Division of Hepato-Gastroenterology, Department of Internal Medicine, Kaohsiung Chang Gung Memorial Hospital, Kaohsiung 833, Taiwan

**Keywords:** albumin–bilirubin grade, Child–Pugh classification A, cutoff, clinical validation, hepatocellular carcinoma, real-world data

## Abstract

**Background:** The albumin–bilirubin (ALBI) grade provides an objective assessment of hepatic reserve, but the need for calculation by means of a formula has hampered its use at the bedside. This study aimed to develop simple cut-off values for ALBI grade and validate its performance in a large multi-center real-world cohort. **Methods:** A mathematical simulation evaluated every possible ALBI pair that falls within the Child–Pugh classification (CP) A range, discretized to 0.1 increments. Cut points for patient stratification without equation-based calculation were derived. Validation was conducted with the Chang Gung Research Database (CGRD), which contains data from 10 hospitals in Taiwan. Patients with same-day albumin and bilirubin measurements in 2024 were included. **Results:** Mathematical modeling identified clinically applicable cutoffs—albumin ≥ 4.4 g/dL or ≤3.5 g/dL and bilirubin ≥ 2.4 mg/dL—with further refinement at albumin 4.0 g/dL and bilirubin ≥ 1.0 mg/dL. Among 7583 CP-A patients, 82% were directly classifiable without computation, with consistent applicability across chronic liver disease and hepatocellular carcinoma (HCC) subgroups. Equation dependence increased only slightly in the HCC group, confirming robustness across disease severities. **Conclusions:** Simplified cutoff rules derived from mathematical modeling and validated in a multi-center cohort enable rapid recognition of ALBI grade in most CP-A patients. This approach enhances the clinical usability of ALBI and supports its integration into patient care, clinical trials, and treatment allocation.

## 1. Introduction

Hepatocellular carcinoma (HCC) is the sixth most common cancer and the third leading cause of cancer-related death worldwide [1]. The majority of HCCs arise from chronic liver disease (CLD) such as chronic hepatitis B or C and steatohepatitis that progress to fibrosis and cirrhosis [2,3]. Liver function has a significant impact on prognosis and treatment allocation, so the evaluation of hepatic functional reserve is the key for prediction of prognosis in HCC [4,5]. The Child–Pugh classification (CP), consisting of five clinical or biochemical parameters, is an extensively used tool for this purpose.

The scoring system was first reported by Child and Turcotte in 1964 to evaluate surgical risk [6]. The modified CP currently in use was introduced in 1973 [7]. Notably, while ascites was originally assessed only by physical examination, the CP system has remained unchanged despite advances in objective imaging modalities such as ultrasonography and computed tomography [8]. In addition, the highest scoring category for serum albumin is “>3.5 g/dL.” Clinically, however, patients with serum albumin of 4.5 g/dL differ significantly from those with 3.5 g/dL, suggesting that this cutoff is insufficiently discriminatory [9]. A similar issue exists for serum bilirubin, as conventional CP cutoffs provide limited prognostic discrimination [10]. In addition, two of the parameters in the CP system—ascites and hepatic encephalopathy—are inherently subjective and may not provide unequivocal assessments [11].

Although HCC represents a clinically critical setting in which liver functional stratification directly determines therapeutic options, assessment of hepatic reserve is fundamentally applicable across the entire spectrum of CLD. Since the majority of treatment modalities are restricted to patients with CP-A, more refined stratification within this subgroup is needed. The Albumin–Bilirubin (ALBI) grade, introduced in 2015, encompasses only two laboratory variables—serum albumin and bilirubin, calculated as continuous variables using a validated formula. The ALBI grade has excellent discrimination and prognostic ability for evaluating hepatic reserve, from which treatment stratification can be derived in both clinical practice and clinical research [12,13]. Recent international guidelines have also endorsed the use of ALBI grade as an objective tool to complement CP in assessing hepatic reserve and to support treatment stratification in both clinical practice and research settings [14,15,16]. Nevertheless, the calculation is not user-friendly in routine practice, despite the availability of online calculators.

It would be very useful for the practicing physician if there were an easy and practical way to determine ALBI grade at a glance. The aim of the current study was to assess whether simplified cutoff rules derived from mathematical simulation could be validated in a large real-world cohort of CP-A patients.

## 2. Materials and Methods

### 2.1. Mathematical Derivation

A deterministic and exhaustive mathematical simulation was carried out to explore the distribution of ALBI grades in all possible ALBI pairs among CP-A status and to determine clinically applicable cutoff values. Laboratory values of albumin and bilirubin were discretized to 0.1-unit increments, and a two-dimensional matrix was constructed, with bilirubin levels plotted on the x-axis and albumin levels on the y-axis. Values indicating B (albumin < 3.0 g/dL or bilirubin > 3.0 mg/dL) were excluded to define the boundaries of the study area, whereas 5.5 g/dL for albumin and 0.2 mg/dL for bilirubin were used as the upper and lower limits, respectively. Combinations of albumin 3.0–3.5 g/dL with bilirubin 2.0–3.0 mg/dL were also excluded, as they correspond to CP-B.

The original ALBI equation was used to derive the ALBI score and grade for each individual ALBI pair. After constructing a two-dimensional matrix and assigning an ALBI grade to each pair, the overall ALBI grade distribution was examined. Distinct and contiguous grading regions were observed across the matrix. The boundaries of these regions were then defined as cutoff values that allow differentiation of ALBI grades at a glance. The predictions made based on these cutoff-based rules were subsequently compared with those obtained from the calculated ALBI grades.

### 2.2. Clinical Validation

To facilitate large-scale validation, data were retrieved from the Chang Gung Research Database (CGRD), which collectively contains medical records of 10 branch hospitals within the Chang Gung Memorial Foundation located in Taiwan [17,18]. All data provided by the CGRD were de-identified and contained only encrypted patient identifiers, with no personally identifiable information accessible to the investigators. Laboratory records were first screened for patients with serum albumin and total bilirubin measured on the same calendar day in 2024. Patient-level deduplication was performed using these encrypted identifiers. For patients with multiple laboratory measurements in 2024, only the most recent record with serum albumin and bilirubin measured on the same date was retained for analysis, ensuring that each patient contributed a single observation. To ensure comparability with the mathematical simulation, only values within the range of 3.0–5.5 g/dL for albumin and 0.3–3.0 mg/dL for bilirubin were analyzed. Patients with albumin < 3.0 g/dL or bilirubin > 3.0 mg/dL, corresponding to CP-B or higher, were excluded.

CLD was defined by International Classification of Diseases, 10th Revision codes for chronic viral hepatitis (B18, B19), alcoholic liver disease (K70), chronic hepatitis (K73), cirrhosis (K74), or hepatocellular carcinoma (C22). Patients having C22 codes were further stratified into the HCC subgroup.

A random sample of 10,000 cases was initially pulled to conduct a preliminary review of data quality and distribution. The ALBI value of each participant was then projected on the preset ALBI matrix established by the mathematical model. Patient distribution within various regions of the ALBI matrix was summarized to facilitate direct comparison with theoretical data and clinical parameters. Cohort differences (CLD and CP subgroups) in distribution were assessed using chi-square goodness-of-fit tests, using the overall cohort distribution as the reference to evaluate the robustness of our simplified cutoffs against clinical shifts in hepatic reserve.

## 3. Results

### 3.1. Mathematical Derivation

Within the defined CP-A range, 700 ALBI pairs were theoretically possible, based on albumin values from 3.1 to 5.5 g/dL (increments of 0.1 g/dL) and bilirubin values from 0.3 to 3.0 mg/dL (0.1 g/dL increments). After exclusion of 55 (5 × 11) pairs (albumin 3.1–3.5 g/dL combined with bilirubin 2.0–3.0 mg/dL) corresponding to CP-B, 645 pairs remained eligible for analysis. By the original ALBI equation, 399 pairs (61.9%) were classified as ALBI grade 1 and 246 pairs (38.1%) as ALBI grade 2.

Given that serum albumin and bilirubin, respectively, reflect hepatic synthetic capacity and excretory function, higher albumin and lower bilirubin levels are indicative of better hepatic reserve. Therefore, ALBI grade 1 corresponds to combinations with favorable values, whereas grade 2 corresponds with unfavorable values relative to the derived thresholds.

All pairs with albumin ≥ 4.4 g/dL were classified as ALBI grade 1 regardless of bilirubin, accounting for 336 pairs (52.1%), whereas all pairs with albumin ≤ 3.5 g/dL were classified as ALBI grade 2 (85 pairs, 13.2%). Pairs with albumin values ≥3.5 and <4.4 g/dL required additional stratification. Within this intermediate albumin range, bilirubin provided further discriminatory power. Fifty-six pairs (8.7%) with bilirubin ≥ 2.4 mg/dL were classified as grade 2. Using these three thresholds (albumin 3.5 and 4.4 g/dL; bilirubin 2.4 mg/dL), a total of 477 of 645 pairs (74.0%) were assigned to grade 1 (*n* = 336) or grade 2 (*n* = 141).

For the remaining 168 pairs (26.0%) within the albumin ≥ 3.5 and <4.4 g/dL g/dL and bilirubin 0.3 to <2.4 mg/dL range, further refinement with albumin 4.0 g/dL and bilirubin 1.0 mg/dL cutoffs provided additional discrimination. Pairs with albumin > 4.0 g/dL and bilirubin < 1.0 mg/dL were classified as ALBI grade 1 (*n* = 21), whereas those with albumin ≤ 4.0 g/dL and bilirubin ≥ 1.0 mg/dL were classified as grade 2 (*n* = 70).

**Figure 1:**
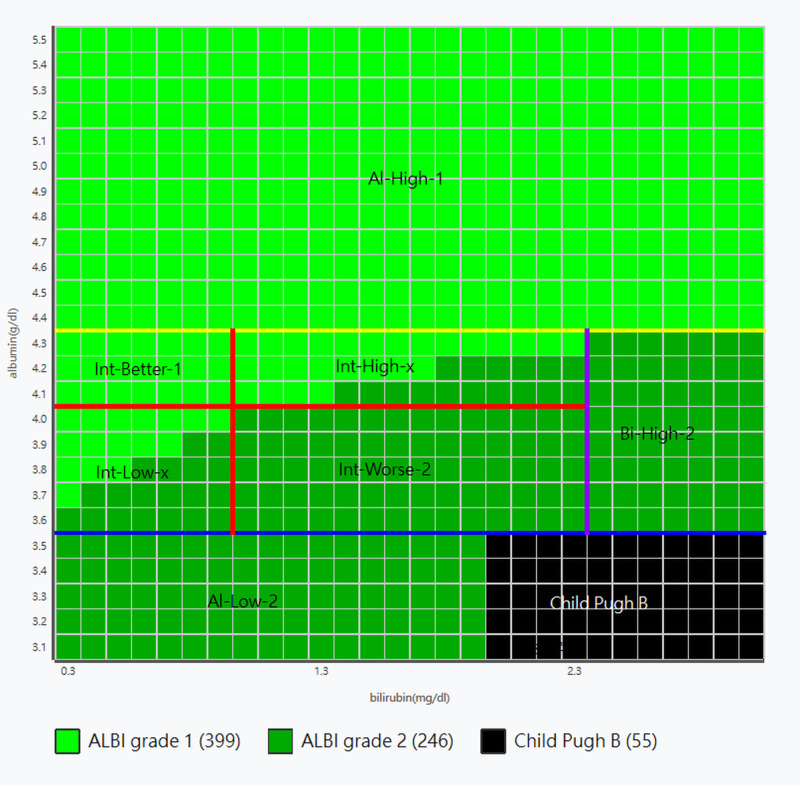
Zonal distribution of albumin–bilirubin (ALBI) pairs within the Child–Pugh classification (CP) A range. Each grid cell represents a theoretical ALBI pair generated from 0.1-increment combinations of albumin (3.1–5.5 g/dL) and bilirubin (0.3–3.0 mg/dL). Cells are color-coded according to ALBI grade assigned by the original formula: light green = grade 1 (*n* = 399); dark green = grade 2 (*n* = 246); and black = CP-B region (*n* = 55). Reference lines indicate simplified cutoff thresholds: albumin 3.5 g/dL (blue), 4.4 g/dL (yellow), bilirubin 2.4 mg/dL (purple), and combined albumin 4.0 g/dL + bilirubin 1.0 mg/dL (red). The intersections of these thresholds define seven practical zones (Al-High-1, Int-Better-1, Int-High-x, Int-Low-x, Int-Worse-2, Al-Low-2, Bi-High-2) used to construct the simplified ALBI classification framework. Abbreviations: ALBI, albumin–bilirubin; Al, albumin; Bi, bilirubin; CP, Child–Pugh classification; Int, intermediate; x, mixed.

**Figure 2:**
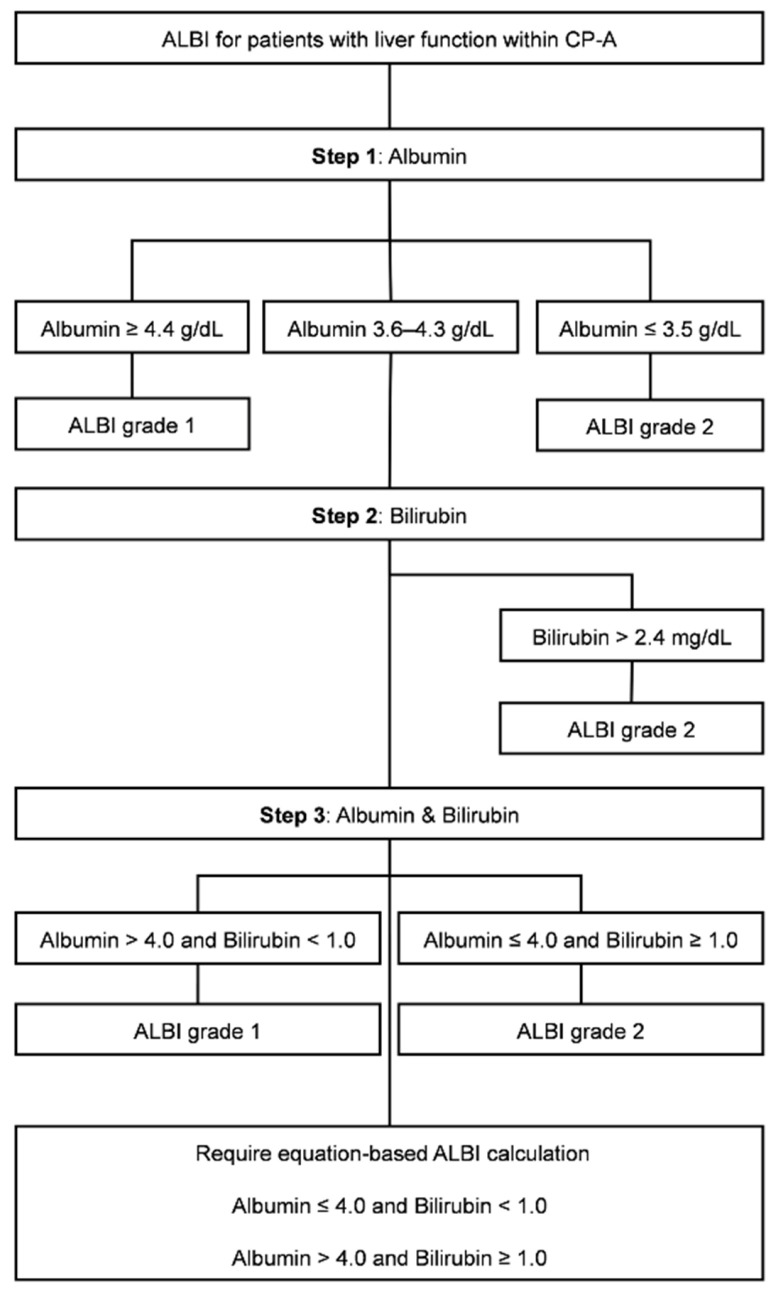
Three-step flowchart for albumin–bilirubin (ALBI) grade classification in Child–Pugh classification (CP) A patients using serum albumin and bilirubin cutoffs. Abbreviations: ALBI, albumin–bilirubin; CP, Child–Pugh Classification.

To facilitate clinical application, the cutoff rules were integrated into a practical framework defined by the intersecting cutoff lines of albumin (3.5, 4.0, and 4.4 g/dL) and bilirubin (1.0 and 2.4 mg/dL), as illustrated in Figure 1. The classification zones were assigned descriptive labels based on their laboratory profiles. In this nomenclature: Al-High denotes high albumin (≥4.4 g/dL); Bil-High denotes high bilirubin (≥2.4 mg/dL); and Int refers to the intermediate albumin range (≥3.5 and <4.4 g/dL). Within the intermediate region, Int-Better-1 indicates favorable liver reserve (defined as albumin > 4.0 g/dL and bilirubin < 1.0 mg/dL), while Int-Worse-2 indicates unfavorable reserve (defined as albumin ≤ 4.0 g/dL and bilirubin ≥ 1.0 mg/dL within this range). Furthermore, Int-High-x identifies the mixed zone with higher albumin and bilirubin (albumin > 4.0 g/dL and bilirubin between 1.0 and <2.4 mg/dL), and Int-Low-x identifies the mixed zone with lower albumin and bilirubin (albumin ≤ 4.0 g/dL and <1.0 mg/dL). The suffix ‘−1’ or ‘−2’ indicates a stable ALBI grade 1 or 2, respectively. The suffix ‘x’ specifically denotes a mixed zone containing both ALBI grade 1 and 2 where equation-based calculation remains mandatory. Detailed definitions and cutoff criteria for each zone are summarized in Supplementary Table S1. These rules were summarized in a three-step algorithm and are shown in Figure 2.

In this primary 0.1-unit simulation, a total of 568 out of 645 eligible pairs (88.1%) were directly classifiable without equation-based calculation. To evaluate the robustness of these cutoffs, a sensitivity analysis was conducted by increasing the simulation step size from 0.1 to 0.2 units. The results remained highly consistent, with the directly classifiable proportion slightly increasing to 89.7% (140/156).

### 3.2. Clinical Validation

**Figure 3:**
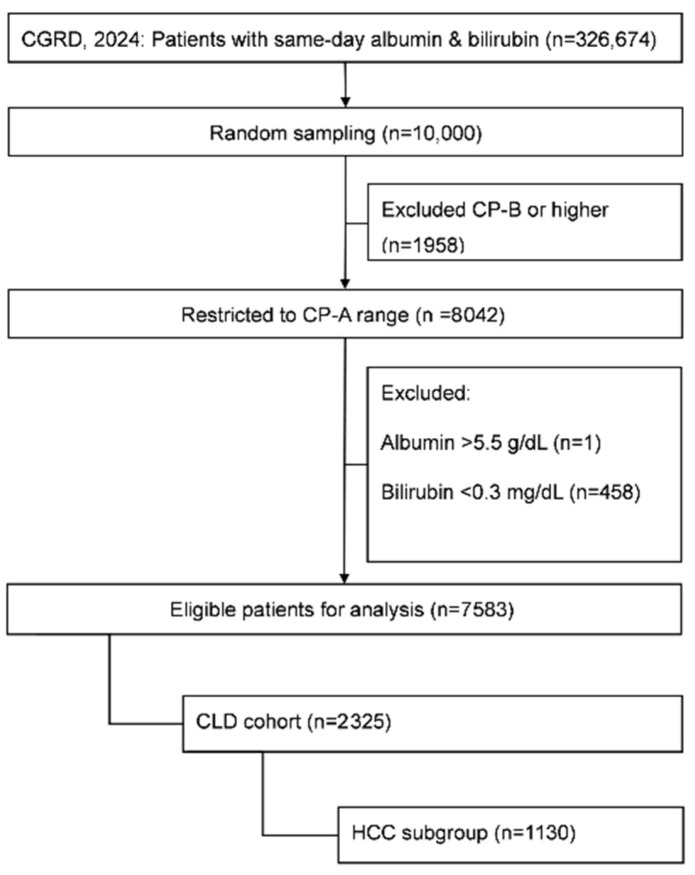
Flowchart of patient selection from the Chang Gung Research Database (CGRD). Abbreviations: CGRD, Chang Gung Research Database; CLD, chronic liver disease; HCC, hepatocellular carcinoma; CP, Child–Pugh classification.

From the CGRD, 326,674 patients had same-day measurements of serum albumin and bilirubin in 2024. To allow comparison with the simulated dataset, a random sample of 10,000 patients was initially selected. After restricting to values within the CP-A range, 8042 patients remained. Exclusion of extreme values (albumin > 5.5 g/dL, *n* = 1; bilirubin = 0.1–0.2 mg/dL, *n* = 458) yielded a final analytic cohort of 7583 patients (94% of CP-A). Of these, 2325 had CLD and 1130 had HCC (Figure 3).

After mapping to the ALBI grids, 5823 patients (76%) fell within the grade 1 region and 1760 (23%) fell within the grade 2 region. As summarized in Table 1, the proportion of grade 2 progressively increased from the total cohort (23%) to CLD (26%) and HCC (30%) subgroups, consistent with their lower albumin and higher bilirubin levels. When stratified into seven labeled regions, the total cohort was distributed as follows: Al-High-1, 3625 (48%); Int-Better-1, 1278 (17%); Int-High-x, 255 (3%); Int-Low-x, 1112 (15%); Int-Worse-2, 282 (4%); Bi- High-2, 39 (1%); and Al-Low-2, 992 (13%). Compared with the mathematical simulation, the real-world cohort showed relatively higher proportions in Int-Better-1 and Int-Low-x, and a lower proportion in Bi-High-2, indicating a clinical shift toward lower albumin and bilirubin combinations. The corresponding distributions in the CLD and HCC subgroups are shown in Table 2. Both subgroups differed significantly from the total cohort (*p* < 0.001 by goodness-of-fit test). Although HCC patients exhibited a higher proportion of ALBI grade 2 (30% vs. 23% in the total cohort), the proportion requiring equation-based calculation increased only slightly (20% vs. 18%).

## 4. Discussion

In this study, we proposed a set of intuitive cutoff values that allow CP-A patients to be visually classified into ALBI grades at a glance using routine laboratory data. Two albumin (≥4.4 g/dL or ≤3.5 g/dL) and one bilirubin (2.4 mg/dL) enable direct classification for most patients, while additional cutoffs at albumin 4.0 g/dL and bilirubin 1.0 mg/dL further distinguish the small intermediate group. These cutoffs facilitate the rapid bedside assessment of hepatic reserve using routine laboratory data. By integrating mathematical derivation and large-scale clinical validation, it was shown that over 80% of CP-A patients could be directly assigned into an ALBI grade without the need for equation-based calculation, delivering a strategy bridging between theoretical rigor and real-world applicability. Notably, even in HCC patients who generally have worse hepatic reserve and a higher proportion of ALBI grade 2, there is only a marginal (2%) increase in the need for equation-based calculation, suggesting that the simplified cutoff rules can remain reliable and clinically convenient regardless of disease severity.

The comparison between the mathematical simulation and real data also demonstrated that the frequency distribution of individual ALBI dimensions did not distribute uniformly among the seven labeled regions. Not all the patterns generated by theory are equally represented in clinical samples, and this result was supported by goodness-of-fit testing. Among the overall, CLD and HCC populations, the ALBI grade 2 ratios became steadily higher along with the anticipated spectrum of liver disease severity. Nevertheless, the majority of patients clustered in the Al-High-1 and Al-Low-2, which can be readily identified without calculation. These comprised about 60% of all CP-A cases, and proved that the majority of patients could be identified quickly just by the albumin concentration. As previously mentioned, the real-world cohort showed a tendency toward lower bilirubin distributions, with 1% in Bi-High-2, representing the largest deviation from mathematical simulation. In the intermediate albumin ranges, Int-Better-1 and Int-Low-x accounted for a higher proportion of patients, while Int-Worse-2 and Int-High-x had smaller proportions. These changes in distribution also support the need for clinical validation rather than solely theoretical modeling. The largest intermediate region, Int-Better-1 (17%) was the largest easy-to-classify set and Int-Low-x (15%) the largest group of elements whose estimates required equation-based computation. In contrast, Int-Worse-2, accounting for approximately 4% of patients, could still be readily identified as ALBI grade 2 without calculation.

Together, these results indicate that the simple cutoff model is robust under various clinical scenarios, even in late-stage disease. Importantly, validation in the HCC cohort provides evidence for applicability in a setting where ALBI is already incorporated into international guidelines to guide systemic therapy allocation. The uniform cutoff-based classification in HCC has the potential to be included in treatment decision strategies.

It is noteworthy that serum albumin and bilirubin levels can also be influenced by non-hepatic factors, which may further contribute to the uneven distribution among the seven regions. A common benign metabolic condition, Gilbert syndrome (GS), may cause isolated unconjugated hyperbilirubinemia while albumin levels remain normal. This syndrome affects approximately 10% of the general population [19]. In our study, most patients within an intermediate albumin level had bilirubin within normal range (0.2–1.2 mg/dL). However, a small subset exhibited higher bilirubin levels. The Int-worse-2 (4%), Int-high-x (3%), and Bi-High-2 (1%) regions together accounted for about 8% of patients, which could partly be explained by the presence of GS. In addition, malnutrition or albuminuria may also have contributed to minor variations in the albumin distribution. While these non-hepatic factors may contribute to minor deviations, our findings indicate that they do not materially affect the robustness of the simplified ALBI classification in liver disease populations.

It should be noted that our simplified framework relies on linear cutoffs to approximate the inherently nonlinear ALBI curve. While this deterministic approach does not account for potential interaction effects between albumin and bilirubin beyond what is defined in the original formula, it was designed to maximize clinical utility at the bedside. By designating ‘Mixed Zones’ for values near the mathematical boundary, we ensure that the simplicity of the tool does not compromise diagnostic rigor in borderline cases. The high consistency (>99%) observed in our large-scale validation cohort supports this linear-based regional classification as a robust surrogate for the complex ALBI equation.

The strength of this study includes the integration of mathematical derivation and multi-center real-world validation, the use of laboratory increments (0.1 g/dL for albumin and 0.1 mg/dL for bilirubin) which are consistent with routine reporting standards [20], and the demonstration of steady performance across both CLD and HCC subgroups. These features enhance direct bedside usability and may facilitate the consistent use of ALBI in clinical trials, where standardized and reproducible liver function assessment is essential. Nevertheless, several limitations should be acknowledged. First, although CGRD is multi-center, it represents a single health system in Taiwan [17,18], and results may not be directly generalized to other populations [17,18,21]. The applicability of the proposed cutoff values may be influenced by regional differences in laboratory standards and heterogeneous albumin and/or bilirubin assays between laboratories [22]. Furthermore, as validation was conducted within a single calendar year, the potential impact of short-term variations in clinical practice patterns or referral behaviors specific to this period cannot be entirely excluded. Therefore, caution should be exercised when extrapolating these simplified cutoff rules to healthcare settings with different laboratory reporting conventions or clinical contexts. Second, while our study demonstrated high consistency in grading between the simplified rules and the original ALBI formula, we did not perform a direct comparison of their prognostic performance regarding overall survival or treatment outcomes. Future longitudinal studies are needed to further evaluate the prognostic performance of this simplified classification across different clinical stages of liver disease. Third, our validation relied on a retrospective database, which may be subject to inherent selection biases. Finally, the original formula was still needed in about 20% of the patients, demonstrating that formula-based calculation cannot be entirely substituted by simplified rules.

## 5. Conclusions

This study established and validated a simplified cutoff-based method for classifying CP-A patients into ALBI grades using routine serum albumin and bilirubin values. By deriving explicit thresholds through mathematical modeling and confirming their applicability in a large multi-center cohort, we showed that most patients can be stratified without formal computation. This approach enhances the usability of ALBI in clinical practice, supports its role in patient stratification for clinical trials and treatment allocation, and may serve as a foundation for extending these rules to broader populations in future studies.

## Figures and Tables

**Table 1:** Distribution of ALBI grades and applicability of simplified cutoff rules in the total cohort, chronic liver disease (CLD), and hepatocellular carcinoma (HCC) subgroups.

	Total Cohort	CLD Cohort	HCC Cohort
Eligible patients, *n*	7583	2325	1130
ALBI grade 1, *n* (%)	5823 (77%)	1729 (74%)	788 (70%)
ALBI grade 2, *n* (%)	1760 (23%)	596 (26%)	342 (30%)
Directly classifiable by cutoff, *n* (%)	6216 (82%)	1885 (81%)	899 (80%)
Equation-required zone, *n* (%)	1367 (18%)	440 (19%)	231 (20%)

Abbreviations: ALBI, albumin–bilirubin; CLD, chronic liver disease; HCC, hepatocellular carcinoma.

**Table 2:** Distribution of Child–Pugh classification (CP) A patients across cutoff-defined albumin–bilirubin (ALBI) regions. Values represent the number and percentage of patients in each region defined by simplified cutoffs of albumin and bilirubin.

Region	Mathematical Simulation (*n* = 645), *n* (%)	Total Cohort (*n* = 7583), *n* (%)	CLD Cohort (*n* = 2325), *n* (%)	HCC Cohort (*n* = 1130), *n* (%)
Al-High-1	336 (52%)	3625 (48%)	1083 (47%)	434 (38%)
Al (albumin)-Low-2	85 (13%)	992 (13%)	262 (11%)	144 (13%)
Bi (bilirubin)-High-2	56 (9%)	39 (1%)	26 (1%)	13 (1%)
Int (intermediate)-Better-1	21 (3%)	1278 (17%)	366 (16%)	213 (19%)
Int-Worse-2	70 (11%)	282 (4%)	148 (6%)	95 (8%)
Int-High-x	42 (7%)	255 (3%)	111 (5%)	53 (5%)
Int-Low-x	35 (5%)	1112 (15%)	329 (14%)	178 (16%)

Abbreviations: ALBI, albumin–bilirubin; CLD, chronic liver disease; HCC, hepatocellular carcinoma; CP, Child–Pugh classification; Al, albumin; Bi, bilirubin; Int, intermediate; x, mixed.

## Data Availability

The data presented in this study are available on request from the corresponding author. The data are not publicly available due to ethical and privacy restrictions, as the data were obtained from the CGRD and access requires approval from the Chang Gung Memorial Hospital Research Ethics Committee.

## Supplementary Materials

The following supporting information can be downloaded at https://www.mdpi.com/article/10.3390/diagnostics16030370/s1: Table S1. Unified definition and classification logic for ALBI zones.

## Abbreviations

The following abbreviations are used in this manuscript:

ALBI	Albumin–Bilirubin grade
Al	Albumin
Bi	Bilirubin
Int	Intermediate
x	Mixed
CP	Child–Pugh classification
CLD	Chronic Liver Disease
HCC	Hepatocellular Carcinoma
CGRD	Chang Gung Research Database
IRB	Institutional Review Board

## References

[1] Bray F., Laversanne M., Sung H., Ferlay J., Siegel R.L., Soerjomataram I., Jemal A. (2024). Global cancer statistics 2022: GLOBOCAN estimates of incidence and mortality worldwide for 36 cancers in 185 countries. CA A Cancer J. Clin..

[2] Jindal A., Thadi A., Shailubhai K. (2019). Hepatocellular carcinoma: Etiology and current and future drugs. J. Clin. Exp. Hepatol..

[3] Kanwal F., Khaderi S., Singal A.G., Marrero J.A., Loo N., Asrani S.K., Amos C.I., Thrift A.P., Gu X., Luster M. (2023). Risk factors for HCC in contemporary cohorts of patients with cirrhosis. Hepatology.

[4] Liu P.-H., Hsu C.-Y., Hsia C.-Y., Lee Y.-H., Su C.-W., Huang Y.-H., Lee F.-Y., Lin H.-C., Huo T.-I. (2016). Prognosis of hepatocellular carcinoma: Assessment of eleven staging systems. J. Hepatol..

[5] Bruix J., Llovet J.M. (2002). Prognostic prediction and treatment strategy in hepatocellular carcinoma. Hepatology.

[6] Child C.G., Turcott J.G. (1964). Surgery and Portal Hypertension. The Liver and Portal Hypertension.

[7] Pugh R., Murray-Lyon I., Dawson J., Pietroni M., Williams R. (1973). Transection of the oesophagus for bleeding oesophageal varices. Br. J. Surg..

[8] Moore K.P., Aithal G.P. (2006). Guidelines on the management of ascites in cirrhosis. Gut.

[9] Carr B.I., Guerra V. (2017). Serum albumin levels in relation to tumor parameters in hepatocellular carcinoma patients. Int. J. Biol. Markers.

[10] Jalan R., Saliba F., Pavesi M., Amoros A., Moreau R., Ginès P., Levesque E., Durand F., Angeli P., Caraceni P. (2014). Development and validation of a prognostic score to predict mortality in patients with acute-on-chronic liver failure. J. Hepatol..

[11] Cholongitas E., Papatheodoridis G., Vangeli M., Terreni N., Patch D., Burroughs A. (2005). Systematic review: The model for end-stage liver disease–should it replace Child-Pugh’s classification for assessing prognosis in cirrhosis?. Aliment. Pharmacol. Ther..

[12] Johnson P.J., Berhane S., Kagebayashi C., Satomura S., Teng M., Reeves H.L., O’Beirne J., Fox R., Skowronska A., Palmer D. (2015). Assessment of liver function in patients with hepatocellular carcinoma: A new evidence-based approach—The ALBI grade. J. Clin. Oncol..

[13] Demirtas C.O., D’Alessio A., Rimassa L., Sharma R., Pinato D.J. (2021). ALBI grade: Evidence for an improved model for liver functional estimation in patients with hepatocellular carcinoma. JHEP Rep..

[14] Singal A.G., Llovet J.M., Yarchoan M., Mehta N., Heimbach J.K., Dawson L.A., Jou J.H., Kulik L.M., Agopian V.G., Marrero J.A. (2023). AASLD Practice Guidance on prevention, diagnosis, and treatment of hepatocellular carcinoma. Hepatology.

[15] Lau G., Obi S., Zhou J., Tateishi R., Qin S., Zhao H., Otsuka M., Ogasawara S., George J., Chow P.K. (2024). APASL clinical practice guidelines on systemic therapy for hepatocellular carcinoma-2024. Hepatol. Int..

[16] Sangro B., Argemi J., Ronot M., Paradis V., Meyer T., Mazzaferro V., Jepsen P., Golfieri R., Galle P., Dawson L. (2025). EASL Clinical Practice Guidelines on the management of hepatocellular carcinoma. J. Hepatol..

[17] Shao S.C., Chan Y.Y., Kao Yang Y.H., Lin S.J., Hung M.J., Chien R.N., Lai C.C., Lai E.C.C. (2019). The Chang Gung Research Database—A multi-institutional electronic medical records database for real-world epidemiological studies in Taiwan. Pharmacoepidemiol. Drug Saf..

[18] Tsai M.-S., Lin M.-H., Lee C.-P., Yang Y.-H., Chen W.-C., Chang G.-H., Tsai Y.-T., Chen P.-C., Tsai Y.-H. (2017). Chang Gung Research Database: A multi-institutional database consisting of original medical records. Biomed. J..

[19] Vítek L., Tiribelli C. (2023). Gilbert’s syndrome revisited. J. Hepatol..

[20] Burtis C.A., Bruns D.E. (2014). Tietz Fundamentals of Clinical Chemistry and Molecular Diagnostics.

[21] Hsieh C.-Y., Su C.-C., Shao S.-C., Sung S.-F., Lin S.-J., Kao Yang Y.-H., Lai E.C.-C. (2019). Taiwan’s national health insurance research database: Past and future. Clin. Epidemiol..

[22] Farrell C.-J.L., Carter A.C. (2016). Serum indices: Managing assay interference. Ann. Clin. Biochem..

