# Supplementary material for: Recognizing ALBI Grade in Child-Pugh A Patients at a Glance: Mathematical Simulation and Large-Scale Clinical Validation
Source: Diagnostics (Basel). 2026 Jan 23;16(3):370. doi: 10.3390/diagnostics16030370 (PMC12896421; doi:10.3390/diagnostics16030370)
Supplement: Supplementary file 1 [file diagnostics-16-00370-s001.zip › diagnostics-4046091-supplementary.pdf]

Table S1. Unified Definition and Classification Logic for ALBI Zones.

Zone Label (New)	Albumin (g/dL)	Bilirubin (mg/dL)	Simplified Logic	Equation Required or Not	Patient Count n (%)
Al-High-1	≥ 4.4	Any	Stable grade 1	No	3,625 (48%)
Al-Low-2	≤ 3.5	Any	Stable grade 2	No	992 (13%)
Bi-High-2	≥ 3.5 and < 4.4	≥ 2.4	Extremely high bilirubin	No	39 (1%)
Int-Better-1	> 4.0 and < 4.4	< 1.0	Favorable liver reserve	No	1,278 (17%)
Int-Worse-2	≥ 3.5 and ≤ 4.0	≥ 1.0	Unfavorable liver reserve	No	282 (4%)
Int-High-x	> 4.0 and < 4.4	≥ 1.0 and < 2.4	Mixed zone (both higher albumin and bilirubin)	Yes	255 (3%)
Int-Low-x	≥ 3.5 and ≤ 4.0	<1.0	Mixed zone (both lower albumin and bilirubin)	Yes	1,112 (15%)

Abbreviations: ALBI, albumin–bilirubin; Al, albumin; Bi, bilirubin; Int, intermediate; x, mixed
